# A novel *Sugarcane bacilliform virus* promoter confers gene expression preferentially in the vascular bundle and storage parenchyma of the sugarcane culm

**DOI:** 10.1186/s13068-017-0850-9

**Published:** 2017-07-04

**Authors:** San-Ji Gao, Mona B. Damaj, Jong-Won Park, Xiao-Bin Wu, Sheng-Ren Sun, Ru-Kai Chen, T. Erik Mirkov

**Affiliations:** 1 National Engineering Research Center for Sugarcane, Fujian Agriculture and Forestry University, Fuzhou, 350002 Fujian China; 2 Texas A&M AgriLife Research, Weslaco, TX 78596 USA; 3 Guangdong Key Lab of Sugarcane Improvement & Biorefinery, Guangzhou Sugarcane Industry Research Institute, Guangzhou, 510316 Guangdong China

**Keywords:** *Sugarcane bacilliform virus* promoter, Tissue-regulated expression, Culm preferential expression, Storage parenchyma, Vascular bundle, *Saccharum* spp. hybrids

## Abstract

**Background:**

*Saccharum* species such as sugarcane and energy cane are key players in the expanding bioeconomy for sugars, bioenergy, and production of high-value proteins. Genomic tools such as culm-regulated promoters would be of great value in terms of improving biomass characteristics through enhanced carbon metabolism for sugar accumulation and/or fiber content for biofuel feedstock. Unlike the situation in dicots, monocot promoters currently used are limited and mostly derived from highly expressed constitutive plant genes and viruses. In this study, a novel promoter region of *Sugarcane bacilliform virus* (SCBV; genus *Badnavirus*, family *Caulimoviridae*), *SCBV21* was cloned and mapped by deletion analysis and functionally characterized transiently in monocot and dicot species and stably in sugarcane.

**Results:**

In silico analysis of *SCBV21* [1816 base pair (bp)] identified two putative promoter regions (PPR1 and PPR2) with transcription start sites (TSS1 and TSS2) and two TATA-boxes (TATAAAT and ATATAA), and several vascular-specific and regulatory elements. Deletion analysis revealed that the 710 bp region spanning PPR2 (with TSS2 and ATATAA) at the 3′ end of *SCBV21* retained the full promoter activity in both dicots and monocots, as shown by transient expression of the *enhanced yellow fluorescent protein* (*EYFP*) gene. In sugarcane young leaf segments, *SCBV21* directed a 1.8- and 2.4-fold higher transient *EYFP* expression than the common maize *ubiquitin 1* (*Ubi1*) and *Cauliflower mosaic virus* 35S promoters, respectively. In transgenic sugarcane, *SCBV21* conferred a preferential expression of the *β-glucuronidase* (*GUS*) gene in leaves and culms and specifically in the culm storage parenchyma surrounding the vascular bundle and in vascular phloem cells. Among the transgenic events and tissues characterized in this study, the *SCBV21* promoter frequently produced higher GUS activity than the *Ubi1* or 35S promoters in a manner that was not obviously correlated with the transgene copy number.

**Conclusions:**

The newly developed plant viral *SCBV21* promoter is distinct from the few existing *SCBV* promoters in its sequence and expression pattern. The potential of *SCBV21* as a tissue-regulated promoter with a strong activity in the culm vascular bundle and its storage parenchyma makes it useful in sugarcane engineering for improved carbon metabolism, increased bioenergy production, and enhanced stress tolerance.

**Electronic supplementary material:**

The online version of this article (doi:10.1186/s13068-017-0850-9) contains supplementary material, which is available to authorized users.

## Background

The development of genomic tools such as promoters that differ in their ability to regulate the temporal and spatial expression patterns of transgenes constitute a major priority for the genetic improvement of major crops and the production of new products at levels useful for commercialization. The use of promoters with different expression patterns is particularly desirable to minimize the risk of transgene silencing in multigene transformation, routinely applied to achieve more complex, and ambitious phenotypes in transgenic crops [1, 2]. Unlike the situation in dicots, monocot promoters currently used are relatively few and mostly derived from highly expressed constitutive plant genes, such as the *Ubiquitin* (*Ubi*) promoters, maize *Ubi1* [3], sugarcane *ub4* and *ub9* [4], rice *RUBQ2* [5], *Porteresia coarctata Ubi2.3* [6], and *Erianthus arundinaceus* Eriubi D7 [7]. To date, tissue-specific monocot promoters have been developed that target gene expression in leaf and root, but only few are functional in stems. This is a crucial deficit for sugarcane (*Saccharum* spp. hybrids), a major sugar and biomass producer accounting for about 40% of the biofuel production worldwide [8]. Promoters functional in the sugarcane culm include sugarcane *dirigent* and *o*-*methyltransferase* from putative defense and fiber biosynthesis-related genes [9], maize *phosphoenolpyruvate carboxylase* [10, 11], and sugarcane *Loading Stem Gene* [12].

Intergenic regions of plant pararetroviruses (family *Caulimoviridae*) have the potential to be used as promoters and could be exploited in the expression of transgenes in monocots or dicots. These include the various enhanced 35S promoters from the dicot-infecting DNA *Cauliflower mosaic virus* (CaMV) [13] or the promoters of monocot-infecting DNA viruses like *Rice tungro bacilliform virus* (RTBV) [14, 15], *Commelina yellow mottle virus* [16], *Taro bacilliform virus* [17], *Banana streak virus* (BSV) [18], and *Sugarcane bacilliform virus* (SCBV) [19, 20]. Viral promoters derived from monocot-infecting DNA viruses are of particular interest because they tend to confer a tissue-specific gene expression, specifically in the vascular system [14, 16–20].

SCBV (genus *Badnavirus*, family *Caulimoviridae*), serologically related to BSV, have a double-stranded DNA genome of around 7.3–7.9 kilobase pair (kb) in size, encoding three open reading frames (ORFs) whose transcription is directed by a single promoter residing between ORF3 (3′ end) and ORF1 (5′ end) [21–25]. SCBV promoters previously examined are derived from two distinct SCBV species recognized by the International Committee on Taxonomy of Viruses, *Sugarcane bacilliform MO virus* (SCBMOV-MOR) and *Sugarcane bacilliform IM virus* (SCBIMV-QLD), originating from Morocco and Australia, respectively [21, 22, 26, 27]. SCBV isolates display a high degree of variability in their nucleotide (nt) sequence [23, 24, 28, 29], and SCBV promoters confer different patterns of gene expression in various plant species [30]. The natural diversity of SCBV could be exploited to isolate additional SCBV promoters with distinct expression patterns.

Few studies have been directed towards investigating the expression pattern of SCBV promoters in sugarcane. The successful use of such promoters depends to a large extent on overcoming the ability of highly polyploid species such as sugarcane to silence transgenes [31–34]. In this study, we report the development of a novel plant viral promoter, *SCBV21*, isolated from a commercial sugarcane variety (CP72-1210) infected with a Texan SCBV isolate (SCBV-TX) and functionally active in sugarcane. Stable expression analyses demonstrated that *SCBV21* conferred a tissue-regulated gene expression, preferentially in leaves and culms and mainly in the storage parenchyma surrounding the vascular bundle and in vascular phloem and sclerenchyma of the sugarcane culm. It is further shown that *SCBV21* exhibited significantly higher levels of gene expression than the common maize *Ubi1* and CaMV 35S promoters. The value of the *SCBV21* promoter in functional gene analysis and in engineering high-biomass producers such as sugarcane and other monocot species for improved carbon metabolism, enhanced stress tolerance, and bioenergy production, is discussed.

## Methods

### Isolation and sequence analysis of the *SCBV21* promoter

The intergenic region of the viral genome in the family *Caulimoviridae* is a potential promoter region (PPR) [27]. Hence, a set of primers, Prom-F and Prom-R (Additional file 1: Table S1) were designed from the conserved sites flanking the PPR based on multiple alignment of the nucleotide sequences of the two published SCBMOV-MOR and SCBIMV-QLD promoters and an unpublished potential SCBV promoter fragment (~2 kb) (kindly provided by Dr. Guo-Hui Zhou, South China Agricultural University). A 1816-base pair (bp) fragment containing the *SCBV21* promoter was PCR amplified from leaf genomic DNA of commercial sugarcane variety CP72-1210 infected with SCBV-TX isolate, using the Prom-F and Prom-R primers. PCR was performed in a total reaction volume of 20 µL using *Taq* DNA polymerase (NEB BioLabs, Ipswich, MA, USA) following the manufacturer’s recommendation with the cycling conditions: one cycle at 94 °C for 4 min, 35 cycles each at 94 °C for 30 s, 52 °C for 30 s, and 72 °C for 2 min, and one cycle at 72 °C for 5 min. The nt sequence of the amplified *SCBV21* (Additional file 2: Figure S1) was deposited into GenBank under accession number KY031904. The PPR and transcription start site (TSS) of *SCBV21* was identified in silico with Neural Network Promoter Prediction (http://www.fruitfly.org/seq_tools/promoter.html) [35], and putative *cis*-acting elements were predicted by PlantCARE (http://bioinformatics.psb.ugent.be/webtools/plantcare) [36] and PLACE database for plant *cis*-acting regulatory DNA elements (https://sogo.dna.affrc.go.jp/cgi-bin/) [37]. Motifs for plant transcription factors (TFs) associated with phloem or xylem histogenesis were identified in *SCBV21* by the plant TF database PlantTFDB version 4.0 (http://planttfdb.cbi.pku.edu.cn) [38]. Partial reverse transcriptase/ribonuclease H (RT/RNAse H) nt sequences (782 nt) from the *SCBV21* promoter and corresponding regions of 12 SCBV and three BSV genomes from GenBank were aligned with the ClustalW algorithm implemented in MEGA 6.0 [39]. Nucleotide sequences of the promoter regions of *SCBV21*, SCBIMV-QLD, and SCBMOV-MOR were also aligned using the same algorithm. Nucleotide sequence identities were estimated by pair-wise sequence comparison using BioEdit programs [40].

### Expression vectors

The amplified *SCBV21* promoter was subcloned into pGEM-T Easy vector (Promega, Madison, WI, USA) as *SCBV21*/pGEM-T. Three *EYFP* expression vectors were generated with *SCBV21*, *Pr4* [*Ubi1* without heat-shock elements, a deletion of 25 bp (5′-TGGACCCCTCTCGAGAGTTCCGCTC-3′) at the 5′ end of *Ubi1*] and CaMV 35S promoters (Additional file 3: Figure S2). The *SCBV21:EYFP*/pSK vector was produced by cloning the *Sal*Ι/*Nco*Ι-released *SCBV21* fragment of *SCBV21*/pGEM-T as a transcriptional fusion with the *EYFP* gene in the *Sal*Ι/*Nco*Ι-digested CaMV 2×35S:*EYFP*-NOS/pSK (pBluescript) vector [41], replacing the CaMV 2×35S promoter. The *Pr4:EYFP*/pSK construct was assembled by cloning the *Hin*dШ/*Nco*Ι-released *Pr4* fragment of *Pr4:GUS*/pUC19 (Invitrogen, Carlsbad, CA, USA) as a transcriptional fusion with *EYFP* into the *Hin*dШ/*Nco*Ι-digested *EYFP*-NOS/pSK, replacing the CaMV 2×35S promoter. The 35S*:EYFP*-NOS/pSK vector was constructed by cloning the *Hin*dШ/*Bam*HΙ-released CaMV 35S fragment from pBI221 (Clontech, Takara Bio USA, Inc., Mountain View, CA, USA) as a transcriptional fusion with the *EYFP* gene in the *Ubi1*:*EYFP*-NOS/pSK vector [41] after digestion with two sets of restriction enzymes, *Bam*HΙ and *Eco*RΙ, and *Eco*RΙ and *Hin*dШ, replacing the *Ubi1* promoter.

Three *GUS* expression vectors were generated with *SCBV21*, *Ubi1*, and CaMV 35S promoters. The *SCBV21:GUS*/pUC19 was constructed by cloning the *Not*Ι-released *SCBV21* fragment from *SCBV21*/pGEM-T as a transcriptional fusion with the *GUS* gene to the *Sph*Ι/*Xba*Ι-digested and blunt ended pBI221, replacing the CaMV 35S promoter. *Ubi1:GUS*/pUC19 (pAHC27) [3] and pBI221 (CaMV 35S:*GUS*) were used.

A series of *SCBV21* deletion constructs were generated from *SCBV21*:*EYFP*-NOS/pSK, using the three restriction enzymes *Xho*I, *Nco*I, and *Stu*I. *Xho*I and *Nco*I sites were incorporated at the 5′ end of forward (SCBV-MF1 and SCBV-MF2) and reverse (SCBV-MR1 and SCBV-MR2) primers, respectively (Additional file 1: Table S1). Deletion fragment ∆nt1014–nt1837 (deletion A) was generated by deleting the region between *Sut*I and *Nco*I in *SCBV21*:*EYFP*-NOS/pSK, followed by blunt ending using the Klenow enzyme (NEB BioLabs). Deletion fragments ∆nt1–nt1010 (deletion B), ∆nt1–nt1105 (deletion C), ∆nt1–nt1010 and ∆nt1732–nt1837 (deletion D), and ∆nt1–nt1105 and ∆nt1732–nt1837 (deletion E) were PCR amplified from *SCBV21*:*EYFP*-NOS/pSK using the four sets of primers MF1/MR1, MF2/MR1, MF1/MR2, and MF2/MR2, respectively, and cloned in the blunt ended *EYFP*-NOS/pSK, replacing full-length *SCBV21*. All constructs were sequenced prior to further use to ensure integrity.

### Preparation of target tissue

Transient *EYFP* and *GUS* gene expression assays were performed on sugarcane (*Saccharum* spp. hybrids) tissues (young leaf, top culm, and root), sweet sorghum (*Sorghum vulgare*) leaves, tobacco (*Nicotiana benthamiana*) leaves, and lima bean (*Phaseolus lunatus* L.) cotyledons. Sugarcane young leaf segment, leaf roll and top culm were collected from field-grown varieties CP72-1210 and CP84-1198 and prepared as previously described [34, 41]. Leaf segments and rolls were cultured on MS0.6 medium [42, 43] for 3–4 and 7–10 days in the dark before transformation, respectively. The top young shoot culms were excised approximately 1 cm thick and used immediately. Roots, collected from 3 month-old greenhouse-grown plants, were sterilized in 10% (v/v) commercial bleach for 20 min and rinsed three times with sterile water before transformation.

Young leaf segments of field-grown sweet sorghum were prepared the same way as sugarcane [34, 41] and incubated on MS0.6 medium for 3–4 days in the dark prior to transformation. *N. benthamiana* seeds were sterilized and germinated on MS medium for 1–2 months before seedling leaves were excised and transformed. Cotyledonary tissue from germinating lima bean seeds was prepared according to Chiera et al. [44]. Briefly, seeds were sterilized in 10% (v/v) commercial bleach for 20 min, washed three times with sterile water, and kept in Magenta GA7 containers between layers of a folded white paper towel saturated with sterile water (25 mL) for 4 days at 26 ± 1 °C under 16 h of illumination (40 µEm^−2^/s). The light green cotyledons were excised from the germinating seedlings prior to transformation.

### Plant transformation and generation of transgenics

All tissues were incubated on MSO medium (MS0.6 with 36.44 g/L d-mannitol and 36.44 g/L d-sorbitol) prior to transformation by particle bombardment. DNA coating for bombardment was performed according to Beyene et al. [41]. Briefly, tungsten particles (1.1 µm, Bio-Rad Laboratories, Hercules, CA, USA) (1 mg) were coated separately with plasmid DNA (1.0 µg) of different constructs using calcium chloride (33.4 µL of 2.5 M) and spermidine (13.4 µL of 0.1 M). A total of 4 μL of the DNA particle suspension (0.5 µg of plasmid DNA per bombardment) was placed in the center of a syringe filter and delivered into tissue with a particle inflow gun using a 1100 psi rupture disk, 26 in. Hg vacuum and 7 cm target distance. Bombarded tissue was maintained on MS0.6 medium at 26 ± 1 °C in the dark until analysis.

For stable gene expression, *Ubi1:bar* (pAHC20) [3] was co-bombarded with the target construct into leaf roll discs. Following bombardment, leaf roll discs were maintained on MS0.6 medium for 7 days in the dark without selection, and then broken into small pieces and incubated on MS0.6 with Bialaphos (4 mg/L) selection for 2 weeks. Subsequently, resistant calli derived from leaf rolls were placed on MS with kinetin (2 mg/L), naphthalene acetic acid (2 mg/L), and Bialaphos (4 mg/L) for 6–8 weeks under a 16 h light/8 h dark cycle. Shoots were produced and transferred to MS rooting medium containing indole-3-butyric acid (4 mg/L) and Bialaphos (4 mg/L). After 4 weeks, rooted seedlings were transplanted into pots in the greenhouse. Screening of seedlings for presence of the selection marker Bialaphos was done by spraying with the herbicide glufosinate ammonium (11.33%) (15 mL/L). Seedlings that survived were grown in the greenhouse for 2–12 months for further analysis.

### Southern blot analysis

Identification of independent transgenic lines was done by Southern blot analysis. Genomic DNA was isolated from leaves using the SDS method [45]. Genomic DNA (10 µg) was digested overnight with *Hin*dIII, separated on a 1% (w/v) agarose gel and blotted onto a nylon membrane in 0.4 M alkaline solution [46]. A *GUS* probe was generated from *Pr4:GUS*/pUC19 by *Bbs*I/*Sac*I digestion and labeled with [α-^32^P] dCTP using the Random Primers DNA Labeling kit (Invitrogen, Carlsbad, CA). Pre-hybridization, hybridization, washing, and detection of DNA gel blots were conducted as described by Sambrook et al. [47] and Mangwende et al. [48], using Church’s buffer.

### Analysis of β-glucuronidase activity

Histochemical analysis of β-glucuronidase (GUS) activity was performed mainly as described by Jefferson et al. [49], using GUS buffer [0.1% (v/v) Triton X-100 and 50 mM sodium phosphate buffer, pH 7.0] with X-Gluc (5-bromo-4-chloro-3-indolyl-β-d-glucuronic acid) (1.0 mM, dissolved in DMF) and the oxidation catalysts, potassium ferricyanide, and potassium ferrocyanide (0.5 mM each, dissolved in 10 mM EDTA, pH 8.0). Stained plant tissues were photographed with a zoom stereomicroscope (Olympus SZX7, Olympus, Center Valley, PA, USA). Quantitative GUS activity was carried out using 4-methylumbelliferyl-β-d-glucuronide (Rose Scientific Ltd., Alberta, Canada) [34, 49]). Fluorescence (emission of 455 nm and excitation of 365 nm) was measured with a VersaFluor (Bio-Rad Laboratories). Protein concentrations were determined with the Bio-Rad protein assay kit.

### Evaluation of *EYFP* expression

Images of different tissues expressing *EYFP* were collected at 48 h post-DNA bombardment using a stereomicroscope (Olympus SZX7, Olympus) fitted with YFPHQ filters (excitation of 490–500 nm and emission of 515–560 nm) and a DP71 digital camera (Olympus). Colored RGB images (4080 × 3072 pixels) of leaf segments were collected using the same stereomicroscope (15×). *EYFP* expression analysis was quantified using the ImageJ software (Rasband 1997–2009) as described by Chiera et al. [44]. The detailed protocol was provided by Gao et al. [34].

### Statistical analysis

The GLM procedure of Statistical Analysis System (8.0 version, SAS Institute, USA) was used for statistical analysis. Multiple comparisons of the means were conducted by the Student–Newman–Keuls (SNK) Test. Pearson correlation analysis (SAS software) was performed on GUS activity and *GUS* copy number (as identified by Southern blot analysis) of the generated GUS transgenic lines.

## Results

### Sequence analysis of *SCBV21*

**Fig. 1:**
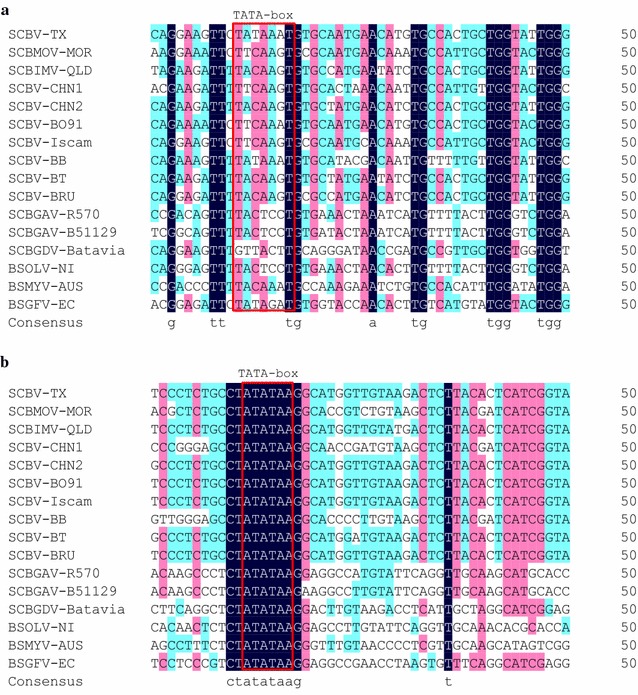
Multiple nucleotide sequence alignments of *SCBV21* [two potential promoter regions (PPRs)], 12 SCBV, and three BSV published isolates using DNAMAN 8.0. The sequence of isolate SCBV-TX (KY031904) was determined in this study, while sequences of isolates SCBMOV-MOR (NC_008017), SCBIMV-QLD (NC_003031), SCBV-CHN1 (KM214357), SCBV-CHN2 (KM214358), SCBV-BO91 (JN377533), SCBV-Iscam (JN377534), SCBV-BB (JN377535), SCBV-BT (JN377536), SCBV-BRU (JN377537), SCBGAV-R570 (FJ824813), SCBGAV-B51129 (FJ824814), SCBGDV-Batavia (FJ439817), BSOLV-NI (NC_003381), BSMYV-AUS (NC_006955), and BSGFV-EC (NC_007002) were obtained from the GenBank database. **a**, **b** Two PPRs of *SCBV21* were identified by Neural Network Promoter Prediction (NNPP, version 2.2). The two TATA-boxes (TATAAAT and ATATAA) that were predicted by PlantCARE and PLACE databases are indicated in a *red box*. Nucleotides that are highlighted in *black* have the highest percentage identity

The 1826-bp *SCBV21* amplified fragment (from SCBV-TX isolate), located at the 3′ end of the SCBV genome, consisted of partial RT/RNAse H genomic (~0.8 kb near the 5′ end) and ~1.0 kb promoter regions (Additional file 2: Figure S1). In silico analysis of *SCBV21* sequence with Neural Network Promoter Prediction (NNPP, version 2.2) identified two PPRs, PPR1 (1055–1105 nt) with transcription start site TSS1 (Fig. 1a; Additional file 2: Figure S1), and PPR2 (1737–1787 nt) with transcription start site TSS2 (Fig. 1b; Additional file 2: Figure S1). Two TATA-boxes (TATAAAT and ATATAA) were observed in PPR1 and PPR2, respectively. Seven CAAT-box and one CAT-box common *cis*-acting elements related to enhancer elements and meristem expression [50, 51], respectively, were found in *SCBV21* (Table 1).

**Table 1:** Putative regulatory motifs enriched in the *SCBV21* promoter

Motif name and sequence^a^	Occurrence and position of motif^b^	Function
Tissue-specific motifs		Expression in phloem, shoot, root, meristem
ASL-box: CTTTA	2 (844; 1631)	
Plant transcription factor motifs		Biological process phloem or xylem biogenesis
Motif 1: AAAAGGGAGCAAAAGGATTAA	1 (298–318)	
Motif 2: TTGAACGATGATTAT	1 (1288–1302)	
Motif 3: ATAAAGAAGCTAAAGCTGAAT	1 (1252–1272)	
Motif 4: TGAAGAAGGATAAAGAAGCTA	1 (1243–1263)	
Enhancer element motif		Enhancement of gene expression
CAAT-box: CAAT	7 (909; 1075; 1201; 1475; 1511; 1540; 1558)	
Meristem-regulated motif		Meristem-regulated gene expression
CAT-box: CAT	1 (1087)	

^a^Motifs were identified by PlantCARE motif sampler (http://bioinformatics.psb.ugent.be/webtools/plantcare) and PLACE for plant *cis*-acting regulatory DNA elements (https://sogo.dna.affrc.go.jp/cgi-bin/). Plant transcription factor motifs were identified by PlantTFDB version 4.0 (http://planttfdb.cbi.pku.edu.cn)

^b^The motif position is given by the number corresponding to the *SCBV21* promoter nucleotide sequence provided in Additional file 2: Figure S1

Multiple nt sequence alignment of the two PPRs of *SCBV21* and those of 12 SCBV and three BSV published isolates comprising BSGFV-EC, BSMYV-AUS, and BSOLV-NI revealed that the TATAAAT sequence in PPR1 of *SCBV21* was found only in SCBV-TX and SCBV-BB isolates (Fig. 1a), whereas the ATATAA sequence in PPR2 of *SCBV21* was conserved among the different SCBV and BSV isolates (Fig. 1b). Since the difference (>20%) in RT/RNase H nt sequence is used as a species demarcation criterion in the *Badnavirus* genus [27], the pair-wise sequence comparison of the partial RT/RNase H sequences of *SCBV21* and the published SCBV and BSV isolates showed that the RT/RNAse H of *SCBV21* shared only 56.5–88.7 and 56.0–60.0% sequence identities with the 12 SCBV and three BSV published isolates, respectively (Additional file 4: Table S2). Sequence identities of 78.4 and 88.7% were observed between SCBV-TX and SCBMOV-MOR and SCBIMV-IM, respectively, based on the RT/RNAse H analysis (Additional file 4: Table S2). Furthermore, SCBV-TX shared only 76.4 and 61.4% nt sequence identity with the published SCBIMV-QLD and SCBMOV-MOR promoters, respectively, based on analysis of the full promoter regions (~1826 bp) (Additional file 2: Figure S1).

### The *SCBV21* core region is PPR2

**Fig. 2:**
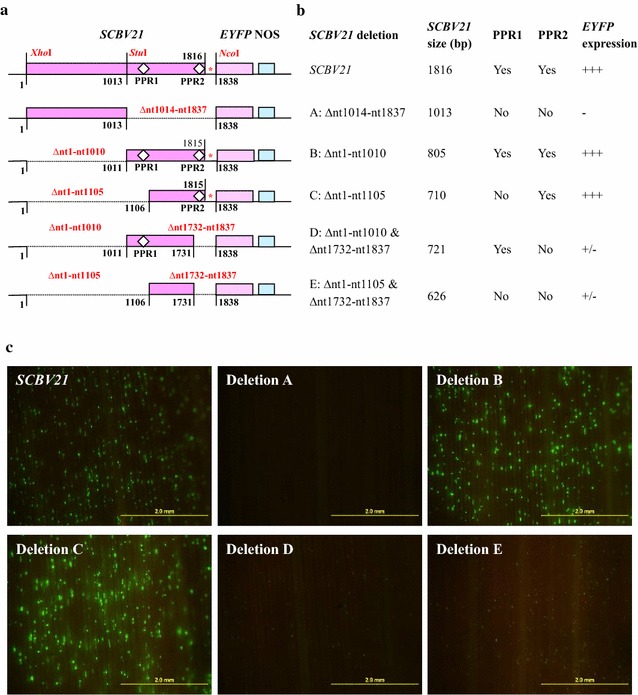
Transient *EYFP* gene expression as directed by *SCBV21* and its deletions in sugarcane. **a** Schematic map of full-length *SCBV21* [1816 base pair (bp)] and its deletions. Nucleotide (nt) 1 is the first nt at the 5′ end of *SCBV21*. Deletions are indicated by *dotted lines* and their nt position of each deletion is indicated *above the dotted line*. The region between *SCBV21* and *EYFP*, marked with an *asterisk* (*) in deletion A, is derived from the multicloning site of pGEM T-Easy vector and is removed from all deletion fragments. In deletions C and D, the guanine base (G) at nt 1816 was deleted during cloning. Three important restriction enzyme sites, *Xho*I, *Stu*I, and *Nco*I used for deletions are marked in deletion A. The two potential promoter regions (PPR1 and PPR2) of *SCBV21,* which are 632 bp apart from each other, are shown with *unfilled square boxes*. The approximate position of primers used to generate deletions is indicated with *filled arrowheads*. **b**, **c** Monitoring of transient *EYFP* expression as directed by *SCBV21* and its deletions in sugarcane young leaf segments. Representative images were collected with a stereomicroscope (Olympus SZX7, Olympus) fitted with YFPHQ filters (excitation of 490–500 nm and emission of 515–560 nm) and a DP71 digital camera (Olympus) (×15 magnification) for 48 h post-DNA bombardment (*scale bar* 2.0 mm). *EYFP* expression levels were scored as high (+++), medium (++), low (+), and none (−), based on the *EYFP* focus count and *EYFP* expression level (mean gray value × pixels)

In order to map an active promoter region within the cloned 1816-bp *SCBV21* fragment, a series of deletions were made around the two putative promoter regions, PPR1 and PPR2 (Fig. 2a). Each deletion was fused to *EYFP* and its promoter activity was tested transiently in sugarcane young leaf segments (Fig. 2b, c). As shown in Fig. 2b, c, a deletion of 824 nt at the 3′ end of *SCBV21* containing PPR1 and PPR2 (deletion A: ∆nt1014–nt1837) abolished its promoter activity. On the other hand, a deletion of 1010 nt at the 5′ end of *SCBV21* (deletion B: ∆nt1–nt1010) with a longer deletion containing PPR1 at the 5′ end of deletion B (deletion C: ∆nt1–nt1105) did not affect the promoter activity. However, deletion of PPR2 located at the 3′ end of *SCBV21* (deletion D: ∆nt1–nt1010 and ∆nt1732–nt1837 and deletion E: ∆nt1–nt1105 and ∆nt1732–nt1837) showed a significant decrease in *EYFP* expression.

### *SCBV21* directs transient *EYFP* gene expression in monocots and dicots

To check if *SCBV21* is active in monocots and dicots, each of *SCBV21*:*EYFP* and *SCBV21*:*GUS* DNA (Additional file 3: Figure S2) was bombarded into leaf roll, stem and root of sugarcane, sweet sorghum young leaf, *N. benthamiana* leaf, and cotyledons of germinating seeds of lima bean. Tissue bombardment experiments demonstrated that *SCBV21* directed *EYFP* and *GUS* gene expression transiently in all tested tissue types (leaves, stems, and roots) in both monocots (sugarcane and sweet sorghum) and dicots (*N*. *benthamiana* and lima bean) (Additional file 5: Figure S3).

**Fig. 3:**
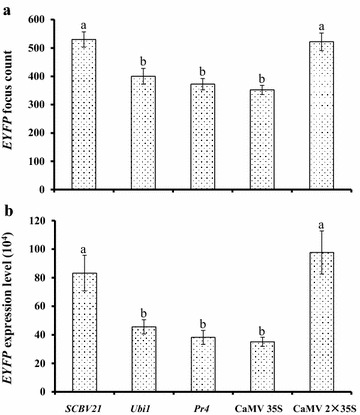
Quantitative assessment of transient *EYFP* gene expression as directed by *SCBV21*, *Ubi1*, *Pr4*, CaMV 35S or enhanced CaMV 35S (2×35S) in sugarcane young leaf segments. Values of **a** *EYFP* focus count and **b** total *EYFP* expression level (10^4^) (mean gray value × pixels) were collected from representative images monitored with a stereomicroscope (Olympus SZX7, Olympus) fitted with YFPHQ filters (excitation of 490–500 nm and emission of 515–560 nm) and a DP71 digital camera (Olympus) (×15 magnification) at 48 h post-DNA bombardment and calculated using ImageJ software as described in “Methods”. Values represent means with standard error from three independent experiments and nine replicates per experiment. Means with the *same letter* are not significantly different at *p* > 0.05

To compare the activity of *SCBV21* with that of four common promoters, CaMV 35S, CaMV 2×35S, *Ubi1*, and *Pr4*, *EYFP* were fused to each promoter (Additional file 3: Figure S2) and its expression was measured in sugarcane young leaf segments post-DNA bombardment (Fig. 3). The kinetics of *EYFP* gene expression revealed that the maximum level of expression was at 48 h post-DNA bombardment (data not shown). Based on the *EYFP* foci count and signal intensity as quantified with ImageJ [34], the activity of *SCBV21* and CaMV 2×35S was significantly (*p* < 0.05) stronger than that of *Ubi1*, *Pr4* and CaMV 35S (Fig. 3). The *EYFP* foci count for *Ubi1*, *Pr4* and CaMV 35S were about 75.5, 70.2 and 66.4% of that of *SCBV21*, and the EYFP expression value was 54.8, 45.9 and 42.2% of that of *SCBV21*, respectively (Fig. 3). However, *EYFP* expression levels driven by *SCBV21* in sugarcane leaf segments were as high as those driven by CaMV 2×35S.

### *SCBV21* directs *GUS* gene expression in a tissue-regulated manner in transgenic sugarcane

Several sugarcane lines transgenic for *SCBV21*:GUS, *Ubi1*:GUS, and CaMV 35S:*GUS* were generated, as identified by Southern blot analysis with a range of *GUS* copy number of 8–14, 13–19, and 4–23, respectively (Table 2). No significant (*p* > 0.05) correlation between GUS activity and *GUS* copy number of the *SCBV21*:GUS, *Ubi1*:GUS, and 35S:GUS lines was found.

**Table 2:** The *SCBV21* promoter drives expression of the *GUS* gene preferentially in the sugarcane culm and leaf

Transgenic line	*GUS* copy number	GUS activity (pmol of 4-methylumbelliferone/min/µg protein)^a^		
		Culm	Leaf	Root
*SCBV21:GUS*	8–14	2649.1 ± 41.3 (2466.3–3252.1)	1260.0 ± 201.8 (1127.9–1384.8)	165.3 ± 4.8 (126.0–233.9)
*Ubi1:GUS*	13–19	23.6 ± 1.5 (18.3–42.5)	46.6 ± 2.6 (31.6–57.4)	58.1 ± 9.0 (37.1–80.1)
35S*:GUS*	4–23	3.8 ± 0.9 (2.0–5.4)	1.3 ± 0.2 (0.4–3.0)	7.2 ± 0.3 (5.5–9.0)
Nontransformed		8.8 ± 0.5 (0.5–13.0)	0.5 ± 0.03 (0.05–0.1)	3.9 ± 0.1 (3.6–4.3)

^a^Average GUS activity was measured in culms, leaves and roots of 1 year-old sugarcane transgenic for *SCBV21*:*GUS*. *Ubi1*:*GUS* and 35S:*GUS* lines were included as a positive control. The number of independent *SCBV21*:*GUS*, *Ubi1*:*GUS* and 35S:*GUS* transgenic lines tested were 5, 5 and 7, respectively. GUS activity represents three technical repetitions and is reported with the standard error. The range of GUS activities for each set of experiments is indicated in parentheses

Quantitative analysis indicated that GUS activity levels of *SCBV21:GUS* sugarcane plants were significantly higher in culms than in leaves and roots (Table 2). Increases in GUS activity of *SCBV21*:*GUS* sugarcane culms were 2.1-fold higher compared to leaves and 16.0-fold higher compared to roots. GUS activity driven by *SCBV21* increased 112-fold in culm and 27.0-fold in leaf compared to that driven by *Ubi1* and 697-fold in culm and 969-fold in leaf, compared to that directed by CaMV 35S (Table 2). GUS activity driven by *SCBV21* was enhanced by 2.8- and 23.0-fold in root compared to that driven by *Ubi1* and CaMV 35S, respectively (Table 2). *SCBV21* conferred high GUS activity in sugarcane culm tissue, irrespective of different spatial positions (top, middle, and bottom) (Table 3) and no significant difference in GUS activity was detected among the *SCBV21*:*GUS* transgenics (Table 3).

**Table 3:** The *SCBV21* promoter drives high levels of *GUS* gene expression in the sugarcane culm

*SCBV21:GUS* transgenic line	GUS activity (pmol of 4-methylumbelliferone/min/µg protein)^a^		
	Top	Middle	Bottom
5A (CP84-1198)	2648.8 ± 27.5	2535.4 ± 48.6	2466. 3 ± 59.3
3501 (CP72-1210)	2560.0 ± 49.4	2500.8 ± 40.4	2574.8 ± 34.6
3512 (CP72-1210)	2668.5 ± 13.1	2668.5 ± 9.9	2688.3 ± 58.9
3515 (CP72-1210)	2653.7 ± 39.5	2569.9 ± 74.6	3252.1 ± 42.2
35123 (CP72-1210)	2693.2 ± 21.5	2651.3 ± 65.3	2604.4 ± 34.6
Nontransformed (CP72-1210)	6.7 ± 0.5	10.4 ± 0.6	9.2 ± 0.4

^a^Average GUS activity was measured in culm top, middle and bottom sections of 1-year-old sugarcane transgenic for *SCBV21*:*GUS*. The number of independent *SCBV21*:*GUS* transgenic lines tested was five (The range of copy number of *GUS* in these lines is 8–14). GUS activity represents six technical repetitions and is reported with the standard error

**Fig. 4:**
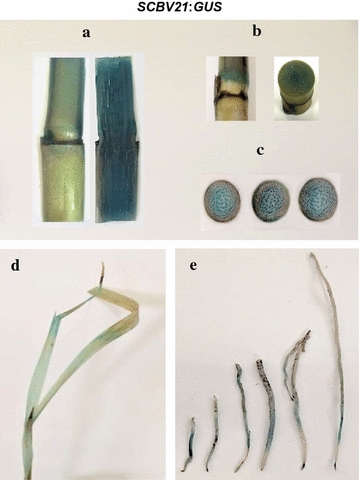
The *SCBV21* promoter directs *GUS* gene expression in a semi-constitutive manner in sugarcane. GUS activity was analyzed histochemically in transgenic sugarcane carrying *SCBV21:GUS* in **a**–**c** culms, **d** leaves and **e** roots. **a** longitudinal culm section, **b** culm with nodes, and **c** transverse culm section

Significant *GUS* expression was histochemically detected in culms, especially in nodes and vascular bundles of transgenic sugarcane carrying *SCBV21:GUS* (Fig. 4a–c). GUS expression was also detected in leaves (Fig. 4d) and root tips (Fig. 4e).

### *SCBV21* confers *GUS* gene expression in the sugarcane culm vascular bundle and storage parenchyma

**Fig. 5:**
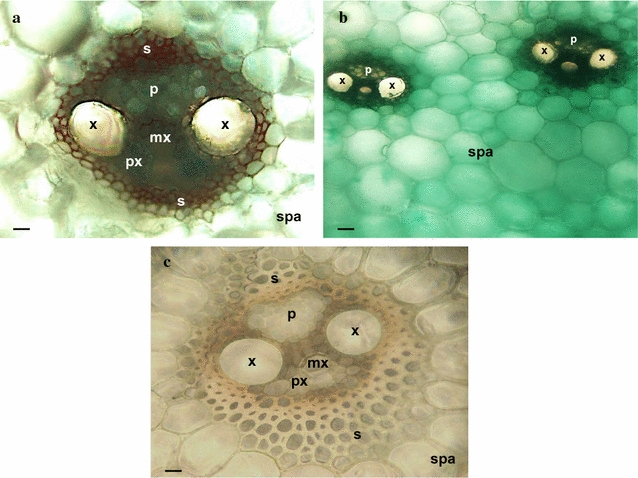
The *SCBV21* promoter directs *GUS* gene expression in the sugarcane culm vasculature and storage parenchyma. GUS activity was analyzed histochemically in transverse sections of **a**, **b** transgenic sugarcane culms carrying *SCBV21*:*GUS*, and in **c** nontransformed sugarcane culms. *x*, xylem; *px*, protoxylem; *mx*, metaxylem; *p*, phloem; *s*, sclerenchyma; *spa*, storage parenchyma. *Scale bar* 50 μm

Histochemical GUS localization of *SCBV21*-driven *GUS* expression revealed that the *SCBV21* promoter conferred vascular *GUS* expression in the culm (Fig. 4), associated with the phloem and sclerenchyma cells of the vascular complex and with the storage parenchymatous tissue surrounding the vascular bundle (Fig. 5a, b). In silico analysis of the *SCBV21* sequence predicted the presence of the ASL-box (CTTTA repeat) [52, 53] and four motifs of plant TFs previously associated with phloem histogenesis [54, 55] (Table 1), with three located at nt 1243–1302 between PPR1 and PPR2 and one in the RT/RNAse H region (Table 1).

## Discussion

We have expanded the repertoire of promoters available for use in monocots by developing a novel plant viral promoter, *SCBV21*, that is preferentially expressed in the culm vascular bundle and the storage parenchyma surrounding the bundle. The activity of *SCBV21* in monocots and dicots was evaluated by adopting a transient gene expression assay, previously shown to be rapid, quantifiable, and reproducible for the comparative analysis of the activity of different promoters [34, 41]. Transient and stable expression analyses using *SCBV21* fusions to *EYFP* and *GUS* genes (*SCBV21:EYFP* and *SCBV21:GUS*) showed that the promoter is functional in both monocots (sugarcane and sweet sorghum) and dicots (*N. benthamiana* and lima bean), consistent with the activity of the *SCBV* promoters from SCBMOV-MOR [19, 20, 30, 56] and SCBIMV-QLD species [57].

### Sequence relatedness of *SCBV21* to other related SCBV and BSV

Similar to other badnaviruses, SCBV are genetically diverse, and the large pool of SCBV variants present in sugarcane is probably due to the vegetative nature of propagation of the host and its long history of movement and cultivation [57]. The extensive genetic diversity of SCBV has been reported in the promoter [57], RT/RNase H [29], and full genomic [25] sequences. In this study, we cloned, mapped, and functionally characterized a novel SCBV promoter, *SCBV21*, in addition to two SCBV promoters previously developed from SCBIMV-QLD [57] and SCBMOV-MOR [19, 20]. *SCBV21* shared low nt sequence identity with the published SCBIMV-QLD and SCBMOV-MOR promoters, respectively based on the full promoter sequence, showing that it is a distinct promoter [58]. *SCBV21* is also different in its RT/RNase H region (872 nt), a common taxonomic marker for species demarcation in the family *Caulimoviridae* [27], since it shared only 56.5–88.7 and 56.0–60.0% nt sequence identity with 12 other SCBV and three BSV published isolates. Furthermore, the *SCBV21* sequence in the genomic intergenic region (a PPR), particularly in the first TATA-box motif showed more divergence than that of the other SCBV and BSV isolates.

### Regulatory region of *SCBV21*

Common core promoter sequences usually contain an initiator and a TATA-box as well as specific *cis*-acting regulatory elements interacting with various enhancers or TFs [2]. Our deletion analysis revealed that the 710-nt region containing PPR2 [with TSS2 and TATA-box (ATATAA)] at the 3′ end of *SCBV21* retained the full promoter activity, suggesting that the RT/RNase H coding region and putative PPR1 [with TSS1 and TATA-box (TATAAAT)] may not be required for *SCBV21* activity. Similarly, previous studies have reported that RT/RNase H was not influenced by important promoter motifs, such as those identified in the SCBMOV-MOR or SCBIMV-QLD promoters [19, 57]. However, SCBMOV-MOR-derived promoters ScBV-1 and ScBV-2, which lacked the TATA-box sequence at PPR2 conferred a decreased *GUS* expression level [19]. Furthermore, deletion of the 5′ end of ScBV-3 promoter did not affect *GUS* expression until it was close to the 254 bp upstream of the TATA-box at PPR2 [59]. The *SCBV839* promoter, derived from SCBIMV-QLD and containing 770 bp upstream of transcript start site (T) conferred a higher GUS activity than *SCBV576* (containing 507 bp prior to T) and *SCBV333* (containing 264 bp prior to T), indicating that putative enhancer sequences are present in the region prior to the TSS [60]. Alternatively, *SCBV537* or *SCBV282*, which contained putative enhancer sequences with no PPR2, increased GUS activity when fused with the truncated maize *alcohol dehydrogenase 1* promoter [60]. These results demonstrate that PPR2 is critical for promoter activity, and some enhancer sequences upstream PPR2 are potential regulatory elements. Alignment of PPR2 sequence of *SCBV21* with those of 12 SCBV and 3 BSV published isolates revealed that the TATA-box (ATATAA) motif has conserved *cis*-acting elements.

### Semi-constitutive gene expression

Histochemical localization of *GUS* expression in situ and quantitative assessment of GUS activity in transgenic sugarcane provides evidence for a higher activity of *SCBV21* in the culm than the leaf and root. The *SCBV21* promoter is different in its semi-constitutive expression pattern from the two previously developed SCBV promoters. For instance, the SCBMOV-MOR promoter was shown to confer high levels of constitutive *GUS* expression in vegetative and reproductive tissues of both monocots (sugarcane, banana, oat, barley, and wheat) and dicots (Arabidopsis and tobacco) [18–20]. However, differences in SCBMOV-MOR promoter specificity were detected among species and tissues, i.e., stronger GUS activity in most tissues of oat and barley than in wheat [30]; and mainly vascular GUS activity in root of banana but constitutive in tobacco [20]. The SCBIMV-QLD promoter was reported to be the strongest in driving reporter gene expression in the leaves, meristems, and roots of glasshouse-grown sugarcane [57]. The *SCBV21* promoter shares more nt sequence homology with the SCBIMV-QLD promoter and, similarly, it is active in leaves and roots and contains several putative meristem-regulated motifs (CAT-box) and enhancer elements (CAAT-box) [50, 51]. However, the activity of *SCBV21* in these tissues is stronger than that of the SCBIMV-QLD promoter, which conferred equal to or non-significantly higher expression levels of the neomycin phosphotransferase II gene than those measured for *Ubi1* in sugarcane [57].

### Preferential gene expression in the culm vascular bundle and storage parenchyma

The vascular-regulated *GUS* expression pattern of *SCBV21* is similar to the one displayed by other badnavirus-derived promoters [14, 16–20]. Some badnaviruses are limited to the vascular tissue like RTBV that replicates only in phloem cells of its host and its promoter drives a strong phloem-specific gene expression [14]. However, other badnaviruses like SCBV, infecting economically important species in the *Poaceae* family, are not phloem-limited [19]; for instance, the expression profile of the *SCBV21* promoter in the vascular bundle, including the phloem cells and the storage parenchyma provides evidence for a more widespread vascular expression than that of RTBV. In addition, *SCBV21* displays a significant strong activity in the culm storage parenchyma, not reported for the existing SCBV promoters from SCBIMV-QLD and SCBMOV-MOR species.

The vascular-regulated activity of *SCBV21* in the culm correlates with the presence of vascular tissue-specific regulatory motifs in its sequence. These motifs include the ASL-box (CTTTA repeat), present in phloem-specific promoters [52, 53] as well as four motifs of plant TFs associated with biological process of phloem histogenesis [54, 55], with three harbored in the PPR1 and PPR2 region. Phloem-regulated expression can be beneficial in imposing a decreased metabolic load on the plant by incorporation of additional phloem-derived cells to ensure proper transport of organic nutrients to cells involved in the reinforcement of the plant axis to counteract the increased weight of the growing plant [61].

The significant *SCBV21*-driven expression in the storage parenchyma of the vascular bundle of the culm is of major importance to the economic value of crops like sugarcane, energy cane, and other high biomass and fiber producers. The economic yield of sugarcane is determined by accumulation of sucrose in the culm, and the sucrose and hexoses are taken up by the storage parenchyma cells [62]. Under conditions favoring sucrose accumulation, the storage parenchyma tissue of sugarcane can store sucrose up to the maximum value of 62% dry weight or 27% fresh weight in theory [62, 63]. *SCBV21* could be advantageous in manipulating certain aspects of sucrose transport and fiber synthesis, and the spatial separation or partitioning between sucrose accumulation and cell wall fiber synthesis. In particular, the *GUS* expression pattern of *SCBV21* is similar to the *green fluorescent protein* expression pattern displayed by the promoter of the cell wall synthesis gene *ShCesA7* in the storage parenchyma of the maturing culm internode of sugarcane [64]. The use of *SCBV21* in co-targeting an elevated expression of primary cell wall synthesis genes (*ShCesA1, ShCesA7, ShCesA9* and *Shbk2l3*) and sugar transporter genes (*ShPST2a*, *ShPST2b*, and *ShSUT4*) [64] in the storage parenchyma would maximize sucrose production and biomass accumulation.

The vascular-regulated expression may be also exploited to develop virus-resistant lines by fusing antiviral constructs to *SCBV21* to control monocot viruses that multiply and translocate in the vascular tissue [65], or to improve plant tolerance to important pests such as the neonate sugarcane borer (*Diatraea saccharalis* F.) larvae by driving the expression of the *Bacillus thuringiensis* δ-endotoxin [66]. *SCBV21* is also potentially useful in other sugarcane biotechnology applications, such as in enhancing the expression of high-value recombinant proteins in the culm of high biomass producers like sugarcane and energy cane [67].

## Conclusions

In this study, a novel plant viral promoter, *SCBV21* (1816 bp) was PCR amplified from the genomic DNA of a commercial sugarcane variety infected with a Texan SCBV isolate, with the aim to expand the repertoire of promoters available for use in monocots such as sugarcane, a major sucrose accumulator and biomass producer. Deletion analysis of *SCBV21* revealed that the 710-nt region containing PPR2 [with TSS2 and TATA-box (ATATAA)] at its 3′ end retained the full promoter activity, suggesting that the RT/RNase H region and putative PPR1 may not be required for *SCBV21* activity. Stable expression analyses demonstrated that *SCBV21* conferred a preferential *GUS* gene expression in the storage parenchyma surrounding the vascular bundle and in vascular phloem and sclerenchyma of the sugarcane culm. It is further shown that *SCBV21* exhibited significantly higher levels of *GUS* gene expression than the common maize *Ubi1* and CaMV 35S promoters. The novel *SCBV21* promoter expression pattern is distinct from that of the few existing *SCBV* promoters in its strong activity in the culm vascular bundle and its storage parenchyma, making it valuable for metabolic engineering to improve plant biomass characteristics through enhanced carbon metabolism for sugar accumulation or increased fiber content for biofuel feedstock.

## Additional files

**Additional file 1: Table S1.** List of primers used for cloning the *SCBV21* promoter and its deletions.

**Additional file 2: Figure S1.** Multiple alignment of nucleotide sequences of the *SCBV21* promoter (KY031904) and the two published SCBIMV-QLD (NC_003031) and SCBMOV-MOR (NC_008017) promoter regions. Two potential promoter regions of *SCBV21* are underlined in red, as identified with Neural Network Promoter Prediction (NNPP, version 2.2). The putative transcription start sites TSS1 and TSS2 within the two regions are marked with an asterisk (*). The two TATA-boxes (TATAAAT and ATATAA) that were predicted by PlantCARE and PLACE databases are indicated in a red box. The partial RT/RNAse H region (782 nucleotides) is indicated in a green box. Nucleotides that are highlighted in black have the highest percentage identity.

**Additional file 3: Figure S2.** Map of promoter:gene constructs used for sugarcane transformation. *SCBV21*: *Sugarcane bacilliform virus* promoter; *Ubi1*: Maize *ubiquitin 1* promoter; *Pr4*: *Ubi1* promoter without heat-shock elements (5′-TGGACCCCTCTCGAGAGTTCCGCTC-3′); E35S: Enhanced *Cauliflower mosaic virus* (CaMV) 35S (2×35S) promoter; *EYFP*: *enhanced yellow fluorescent protein* gene; *GUS*: *β-glucuronidase* gene; Nos: *Agrobacterium tumefaciens* nopaline synthase terminator. *Ubi1:GUS* (pAHC27 vector) [3] and CaMV 35S:*GUS* (pBI221 vector) (Clontech, Takara Bio USA, Inc., Mountain View, CA, USA) are used. B, *Bam*HI; Bb, *Bbs*I; E, *Eco*RI; H, *Hin*dIII; N, *Nco*I; P, *Pst*I; Sa, *Sal*l; Sc, *Sac*I; Sm, *Sma*I; Sp, *Sph*I; X, *Xba*I; Xh, *Xho*I; and Xm, *Xma*I. Unique enzyme sites are indicated in red. Boxes are not drawn to scale. A triangle represents the deletion of heat-shock elements (25 bp) in the *Ubi1* promoter.

**Additional file 4: Table S2.** Identity (%) of nucleotide sequences of partial reverse transcriptase/ribonulcease H (RT/RNAse H) region (872 nt) of *Sugarcane bacilliform virus* (SCBV) promoter (*SCBV21*), and 12 SCBV and three *Banana streak virus* published isolates.

**Additional file 5: Figure S3.** Transient expression of *EYFP* and *GUS* genes as directed by the *SCBV21* promoter in monocot and dicot tissues. **a**–**f** fluorescent images and **g**–**l** color images were collected with a stereomicroscope (Olympus SZX7, Olympus, Center Valley, PA, USA) fitted with YFPHQ filters (excitation of 490–500 nm and emission of 515–560 nm) and a DP71 digital camera (Olympus) (9.5×, 15× or 24× magnification) with YFP filter and blank light, respectively at 48 h post-DNA bombardment with *SCBV21:EYFP* or *SCBV21:GUS* (*scale bar* 1.0 mm). **a** and **g** sugarcane leaf roll disc, **b** and **h** sugarcane culm, **c** and **i** sugarcane root, **d** and **j** sweet sorghum leaf, **e** and **k** tobacco leaf, **f** and **l** lima bean cotyledon.

## Abbreviations

BSV: *Banana streak virus*
CaMV: *Cauliflower mosaic virus*
DMF: *N*,*N*-dimethylformamide
EDTA: ethylenediaminetetraacetic acid
EYFP: enhanced yellow fluorescent protein
GUS: β-glucuronidase
NOS: *Agrobacterium tumefaciens* nopaline synthase
MS: Murashige and Skoog medium
pSK: pBluescript
RT/RNase H: reverse transcriptase/ribonuclease H
PPR: potential promoter region
RTBV: *Rice tungro bacilliform virus*
SCBIMV-QLD: *Sugarcane bacilliform IM virus*-Queensland
SCBMOV-MOR: *Sugarcane bacilliform MO virus*-Morocco
TF: transcription factor
TSS: transcription start site
Ubi1: maize *ubiquitin* 1
X-Gluc: 5-bromo-4-chloro-3-indolyl-β-d-glucuronic acid
