# Supplementary material for: A novel Sugarcane bacilliform virus promoter confers gene expression preferentially in the vascular bundle and storage parenchyma of the sugarcane culm
Source: Biotechnol Biofuels. 2017 Jul 4;10:172. doi: 10.1186/s13068-017-0850-9 (PMC5496340; doi:10.1186/s13068-017-0850-9)
Supplement: Supplementary file 1 — Additional file 1: Table S1. List of primers used for cloning the SCBV21 promoter and its deletions. [file 13068_2017_850_MOESM1_ESM.docx]

**Table S1** **List of primers used for cloning the *SCBV21* promoter and its deletions**

| **Promoter** | **Primer** | **Sequence (5'→3')** |
| --- | --- | --- |
| *SCBV21* | Prom-F | GAAGAACAGCATGCTGAACATCTGTGGAAGATGC |
|  | Prom-R | CAAACTTGCTCAAATGATCATGTGGTGAACTACCGATG |
| *SCBV21* deletion fragment | MF1 | TTACTCGAGGCCTGCATATCAGTTCACATCTGG |
|  | MR1 | TTACCATGGAAACTTGCTCAAATGATCATGTGGTGAACTACC |
|  | MF2 | TTACTCGAGATCTTTTAGAAGAATATGTCCAAGAACG |
|  | MR2 | TTACCATGGCACCAGCCGAGCGCTATGCTTGCGTAG |
