# Supplementary material for: A novel Sugarcane bacilliform virus promoter confers gene expression preferentially in the vascular bundle and storage parenchyma of the sugarcane culm
Source: Biotechnol Biofuels. 2017 Jul 4;10:172. doi: 10.1186/s13068-017-0850-9 (PMC5496340; doi:10.1186/s13068-017-0850-9)
Supplement: Supplementary file 2 — Additional file 2: Figure S1. Multiple alignment of nucleotide sequences of the SCBV21 promoter (KY031904) and the two published SCBIMV-QLD (NC_003031) and SCBMOV-MOR (NC_008017) promoter regions. Two potential promoter regions of SCBV21 are underlined in red, as identified with Neural Network Promoter Prediction (NNPP, version 2.2). The putative transcription start sites TSS1 and TSS2 within the two regions are marked with an asterisk (*). The two TATA-boxes (TATAAAT and ATATAA) that were predicted by PlantCARE and PLACE databases are indicated in a red box. The partial RT/RNAse H region (782 nucleotides) is indicated in a green box. Nucleotides that are highlighted in black have the highest percentage identity. [file 13068_2017_850_MOESM2_ESM.pptx]

## Slide 1
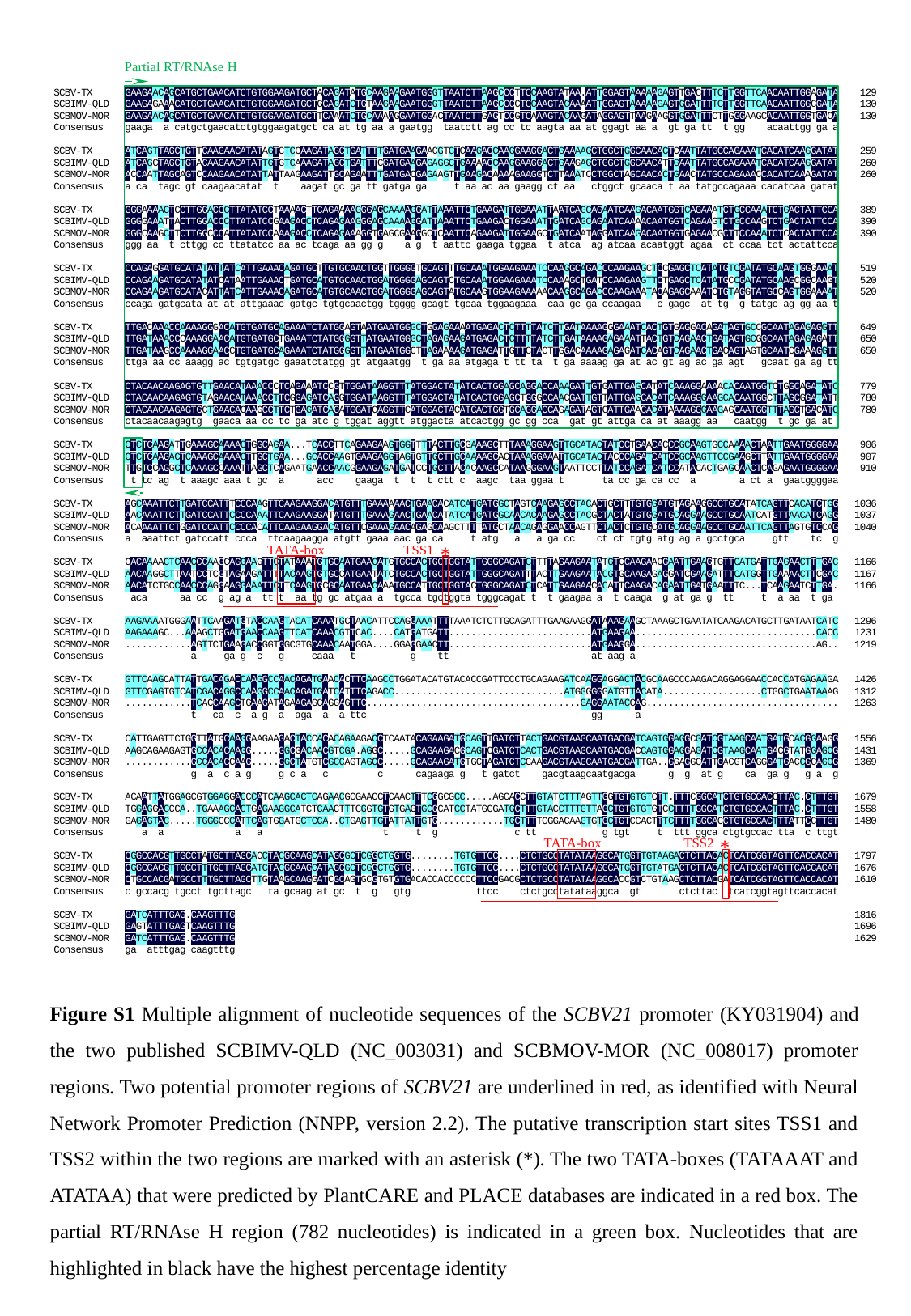

Partial RT/RNAse H
*
TATA-box TSS1
*
TATA-box TSS2
Figure S1 Multiple alignment of nucleotide sequences of the SCBV21 promoter (KY031904) and the two published SCBIMV-QLD (NC_003031) and SCBMOV-MOR (NC_008017) promoter regions. Two potential promoter regions of SCBV21 are underlined in red, as identified with Neural Network Promoter Prediction (NNPP, version 2.2). The putative transcription start sites TSS1 and TSS2 within the two regions are marked with an asterisk (*). The two TATA-boxes (TATAAAT and ATATAA) that were predicted by PlantCARE and PLACE databases are indicated in a red box. The partial RT/RNAse H region (782 nucleotides) is indicated in a green box. Nucleotides that are highlighted in black have the highest percentage identity
