# Supplementary material for: A novel Sugarcane bacilliform virus promoter confers gene expression preferentially in the vascular bundle and storage parenchyma of the sugarcane culm
Source: Biotechnol Biofuels. 2017 Jul 4;10:172. doi: 10.1186/s13068-017-0850-9 (PMC5496340; doi:10.1186/s13068-017-0850-9)
Supplement: Supplementary file 3 — Additional file 3: Figure S2. Map of promoter:gene constructs used for sugarcane transformation. SCBV21: Sugarcane bacilliform virus promoter; Ubi1: Maize ubiquitin 1 promoter; Pr4: Ubi1 promoter without heat-shock elements (5′-TGGACCCCTCTCGAGAGTTCCGCTC-3′); E35S: Enhanced Cauliflower mosaic virus (CaMV) 35S (2×35S) promoter; EYFP: enhanced yellow fluorescent protein gene; GUS: β-glucuronidase gene; Nos: Agrobacterium tumefaciens nopaline synthase terminator. Ubi1:GUS (pAHC27 vector) [3] and CaMV 35S:GUS (pBI221 vector) (Clontech, Takara Bio USA, Inc., Mountain View, CA, USA) are used. B, BamHI; Bb, BbsI; E, EcoRI; H, HindIII; N, NcoI; P, PstI; Sa, Sall; Sc, SacI; Sm, SmaI; Sp, SphI; X, XbaI; Xh, XhoI; and Xm, XmaI. Unique enzyme sites are indicated in red. Boxes are not drawn to scale. A triangle represents the deletion of heat-shock elements (25 bp) in the Ubi1 promoter. [file 13068_2017_850_MOESM3_ESM.pptx]

## Slide 1
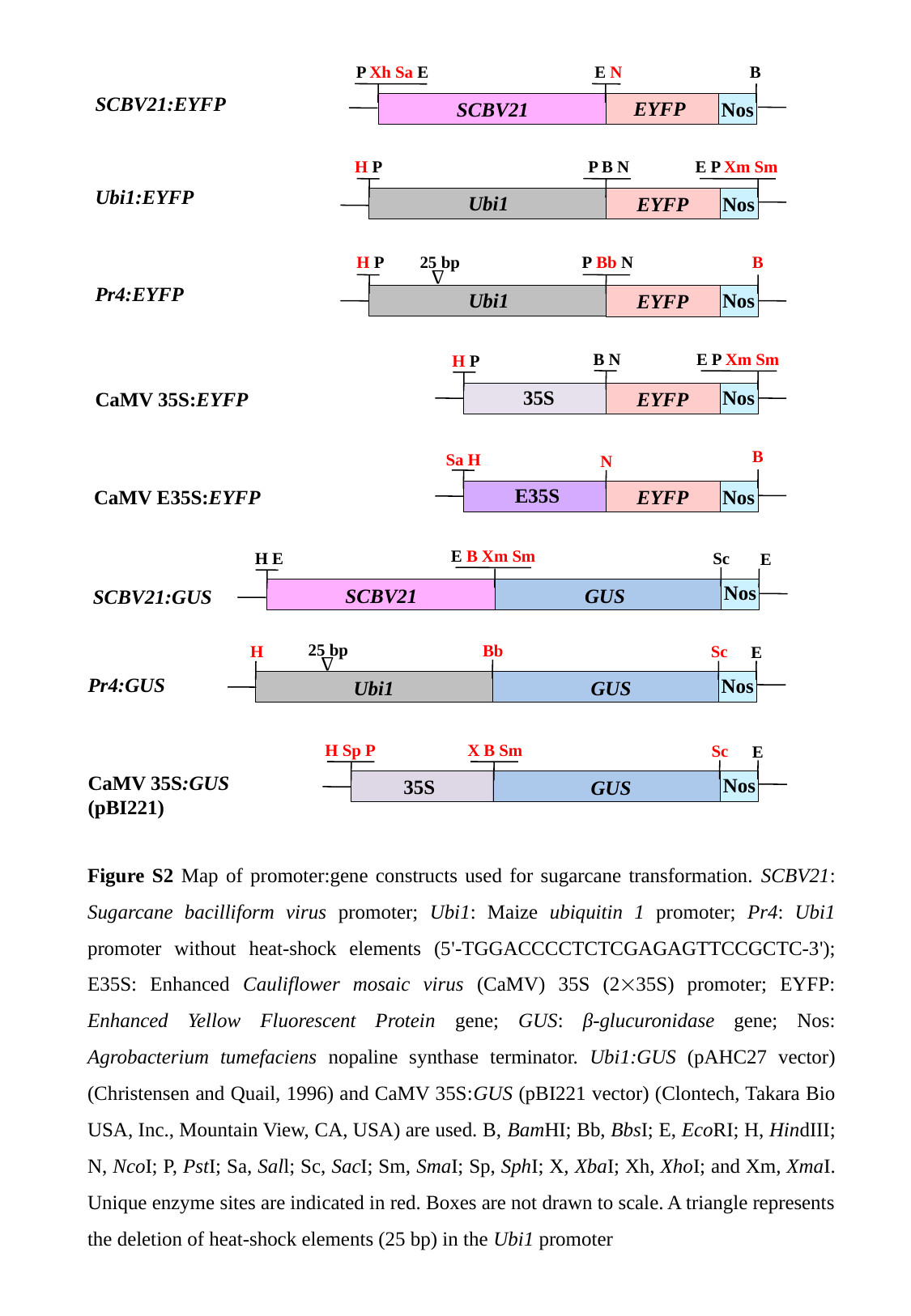

B
P Xh Sa E
E N
EYFP
SCBV21
Nos
H P
P B N
E P Xm Sm
Ubi1
Nos
EYFP
H P
P Bb N
B
Nos
Ubi1
EYFP
B N
E P Xm Sm
H P
Nos
35S
EYFP
B
Sa H
N
E35S
Nos
EYFP
E B Xm Sm
H E
Sc
E
Nos
SCBV21
GUS
Bb
Sc
H
E
Nos
Ubi1
GUS
H Sp P
X B Sm
Sc
E
Nos
35S
GUS
SCBV21:EYFP
Ubi1:EYFP
25 bp
Δ
Pr4:EYFP
CaMV 35S:EYFP
CaMV E35S:EYFP
SCBV21:GUS
25 bp
Δ
Pr4:GUS
CaMV 35S:GUS (pBI221)
Figure S2 Map of promoter:gene constructs used for sugarcane transformation. SCBV21: Sugarcane bacilliform virus promoter; Ubi1: Maize ubiquitin 1 promoter; Pr4: Ubi1 promoter without heat-shock elements (5'-TGGACCCCTCTCGAGAGTTCCGCTC-3'); E35S: Enhanced Cauliflower mosaic virus (CaMV) 35S (2×35S) promoter; EYFP: Enhanced Yellow Fluorescent Protein gene; GUS: β-glucuronidase gene; Nos: Agrobacterium tumefaciens nopaline synthase terminator. Ubi1:GUS (pAHC27 vector) (Christensen and Quail, 1996) and CaMV 35S:GUS (pBI221 vector) (Clontech, Takara Bio USA, Inc., Mountain View, CA, USA) are used. B, BamHI; Bb, BbsI; E, EcoRI; H, HindIII; N, NcoI; P, PstI; Sa, Sall; Sc, SacI; Sm, SmaI; Sp, SphI; X, XbaI; Xh, XhoI; and Xm, XmaI. Unique enzyme sites are indicated in red. Boxes are not drawn to scale. A triangle represents the deletion of heat-shock elements (25 bp) in the Ubi1 promoter
