# Supplementary material for: A novel Sugarcane bacilliform virus promoter confers gene expression preferentially in the vascular bundle and storage parenchyma of the sugarcane culm
Source: Biotechnol Biofuels. 2017 Jul 4;10:172. doi: 10.1186/s13068-017-0850-9 (PMC5496340; doi:10.1186/s13068-017-0850-9)
Supplement: Supplementary file 4 — Additional file 4: Table S2. Identity (%) of nucleotide sequences of partial reverse transcriptase/ribonulcease H (RT/RNAse H) region (872 nt) of Sugarcane bacilliform virus (SCBV) promoter (SCBV21), and 12 SCBV and three Banana streak virus published isolates. [file 13068_2017_850_MOESM4_ESM.docx]

**Table S2** **Identity (%) of nucleotide sequences of partial reverse transcriptase/ribonulcease H (RT/RNAse H) region (872 nt) of *Sugarcane bacilliform virus* (SCBV) promoter (*SCBV21*), and 12 SCBV and three *Banana streak virus* published isolates**

| **No.** | **Isolate ^a^** | **1** | **2** | **3** | **4** | **5** | **6** | **7** | **8** | **9** | **10** | **11** | **12** | **13** | **14** | **15** | **16** |
| --- | --- | --- | --- | --- | --- | --- | --- | --- | --- | --- | --- | --- | --- | --- | --- | --- | --- |
| 1 | SCBV-TX | 100 | 78.4 | 88.7 | 77.1 | 87.1 | 87.3 | 79.1 | 78.2 | 86.5 | 88.7 | 61.3 | 61.1 | 56.5 | 60.0 | 56.4 | 59.5 |
| 2 | SCBMOV-MOR |  | 100 | 79.0 | 78.1 | 79.9 | 77.7 | 93.1 | 81.6 | 79.5 | 78.9 | 62.1 | 60.6 | 58.4 | 59.8 | 57.9 | 59.1 |
| 3 | SCBIMV-QLD |  |  | 100 | 78.7 | 90.0 | 86.5 | 79.4 | 78.2 | 89.9 | 91.8 | 60.5 | 60.2 | 57.8 | 59.6 | 56.0 | 58.2 |
| 4 | SCBV-CHN1 |  |  |  | 100 | 79.9 | 76.3 | 78.7 | 82.8 | 78.7 | 78.0 | 57.5 | 56.5 | 57.0 | 57.3 | 55.3 | 58.1 |
| 5 | SCBV-CHN2 |  |  |  |  | 100 | 86.8 | 80.9 | 80.3 | 92.9 | 91.5 | 60.6 | 60.9 | 58.8 | 61.0 | 55.9 | 58.3 |
| 6 | SCBV-BO91 |  |  |  |  |  | 100 | 79.1 | 78.5 | 87.4 | 88.2 | 59.3 | 59.3 | 57.4 | 59.7 | 56.7 | 58.7 |
| 7 | SCBV-Iscam |  |  |  |  |  |  | 100 | 82.3 | 81.2 | 80.5 | 60.6 | 59.6 | 60.4 | 60.5 | 58.4 | 59.2 |
| 8 | SCBV-BB |  |  |  |  |  |  |  | 100 | 79.9 | 78.7 | 58.6 | 58.7 | 55.5 | 58.6 | 55.6 | 57.0 |
| 9 | SCBV-BT |  |  |  |  |  |  |  |  | 100 | 89.3 | 59.0 | 60.0 | 58.3 | 60.1 | 56.0 | 58.1 |
| 10 | SCBV-BRU |  |  |  |  |  |  |  |  |  | 100 | 60.1 | 60.7 | 56.9 | 59.3 | 56.1 | 58.3 |
| 11 | SCBGAV-R570 |  |  |  |  |  |  |  |  |  |  | 100 | 94.2 | 67.5 | 81.8 | 66.2 | 66.4 |
| 12 | SCBGAV-B51129 |  |  |  |  |  |  |  |  |  |  |  | 100 | 66.4 | 80.9 | 65.5 | 68.0 |
| 13 | SCBGDV-Batavia |  |  |  |  |  |  |  |  |  |  |  |  | 100 | 65.9 | 68.0 | 63.9 |
| 14 | BSOLV-NI |  |  |  |  |  |  |  |  |  |  |  |  |  | 100 | 67.4 | 65.0 |
| 15 | BSMYV-AUS |  |  |  |  |  |  |  |  |  |  |  |  |  |  | 100 | 62.0 |
| 16 | BSGFV-EC |  |  |  |  |  |  |  |  |  |  |  |  |  |  |  | 100 |

**^a^** The RT/RNAse H nucleotide sequence of isolate SCBV-TX (KY031904) was determined in this study, while sequences of isolates SCBMOV-MOR (NC_008017), SCBIMV-QLD (NC_003031), SCBV-CHN1 (KM214357), SCBV-CHN2 (KM214358), SCBV-BO91 (JN377533), SCBV-Iscam (JN377534), SCBV-BB (JN377535), SCBV-BT (JN377536), SCBV-BRU (JN377537), SCBGAV-R570 (FJ824813), SCBGAV-B51129 (FJ824814), SCBGDV-Batavia (FJ439817), BSOLV-NI (NC_003381), BSMYV-AUS (NC_006955) and BSGFV-EC (NC_007002) were obtained from the GenBank database
