# Supplementary material for: A novel Sugarcane bacilliform virus promoter confers gene expression preferentially in the vascular bundle and storage parenchyma of the sugarcane culm
Source: Biotechnol Biofuels. 2017 Jul 4;10:172. doi: 10.1186/s13068-017-0850-9 (PMC5496340; doi:10.1186/s13068-017-0850-9)
Supplement: Supplementary file 5 — Additional file 5: Figure S3. Transient expression of EYFP and GUS genes as directed by the SCBV21 promoter in monocot and dicot tissues. a–f fluorescent images and g–l color images were collected with a stereomicroscope (Olympus SZX7, Olympus, Center Valley, PA, USA) fitted with YFPHQ filters (excitation of 490–500 nm and emission of 515–560 nm) and a DP71 digital camera (Olympus) (9.5×, 15× or 24× magnification) with YFP filter and blank light, respectively at 48 h post-DNA bombardment with SCBV21:EYFP or SCBV21:GUS (scale bar 1.0 mm). a and g sugarcane leaf roll disc, b and h sugarcane culm, c and i sugarcane root, d and j sweet sorghum leaf, e and k tobacco leaf, f and l lima bean cotyledon. [file 13068_2017_850_MOESM5_ESM.pptx]

## Slide 1
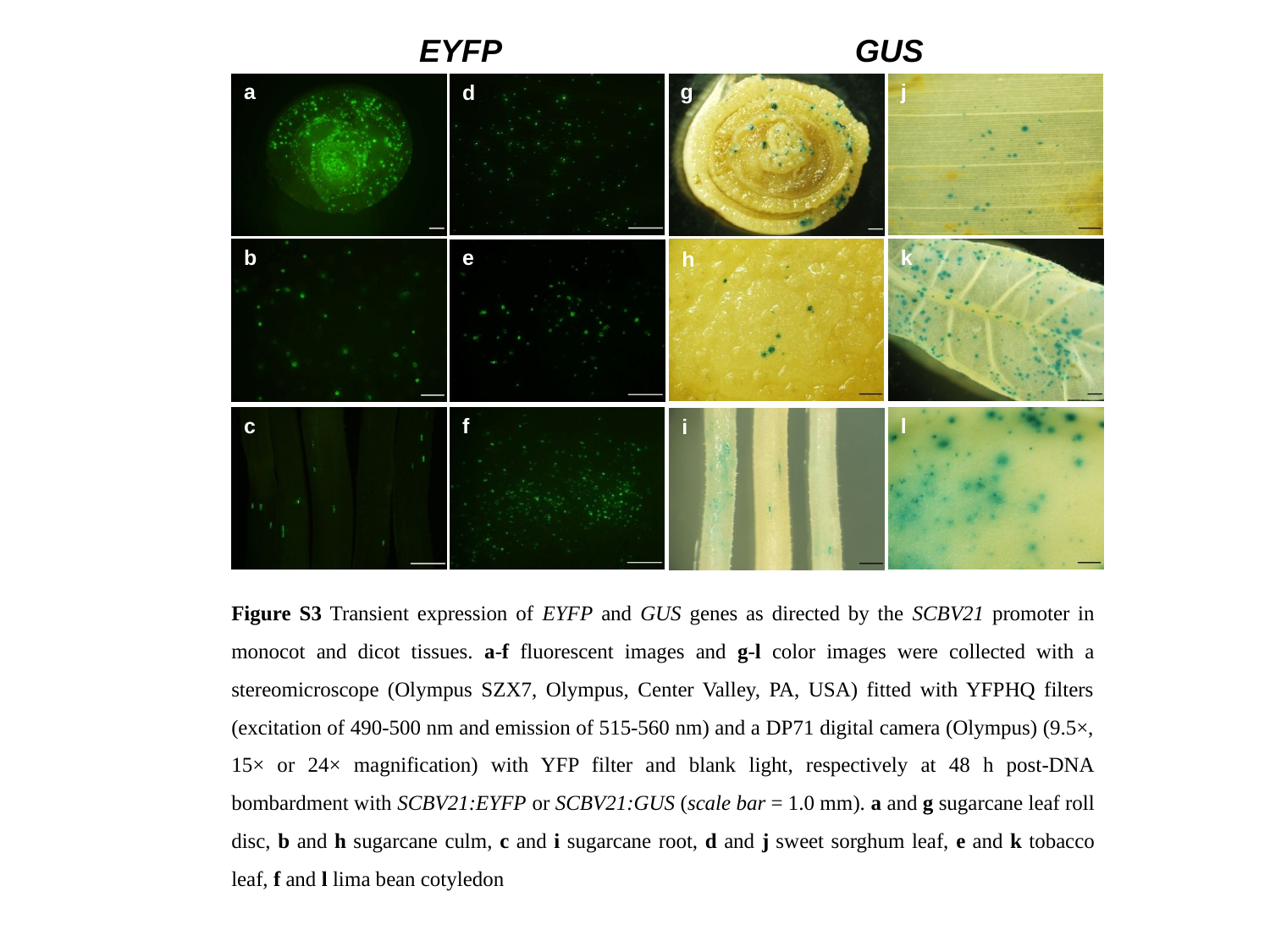

EYFP
GUS
j
a
g
d
k
b
e
h
c
f
l
i
Figure S3 Transient expression of EYFP and GUS genes as directed by the SCBV21 promoter in monocot and dicot tissues. a-f fluorescent images and g-l color images were collected with a stereomicroscope (Olympus SZX7, Olympus, Center Valley, PA, USA) fitted with YFPHQ filters (excitation of 490-500 nm and emission of 515-560 nm) and a DP71 digital camera (Olympus) (9.5×, 15× or 24× magnification) with YFP filter and blank light, respectively at 48 h post-DNA bombardment with SCBV21:EYFP or SCBV21:GUS (scale bar = 1.0 mm). a and g sugarcane leaf roll disc, b and h sugarcane culm, c and i sugarcane root, d and j sweet sorghum leaf, e and k tobacco leaf, f and l lima bean cotyledon
